# A meta-analysis-informed diagnostic stratification tool for invasive pulmonary aspergillosis in severe fever with thrombocytopenia syndrome: systematic review, meta-analysis, and single-center cohort-based assessment

**DOI:** 10.3389/fcimb.2026.1747950

**Published:** 2026-07-02

**Authors:** Ruize Ma, Shuwen Ding, Yanli Xu, Jingxia Wang, Ruihua Zhang, Ziruo Ge, Di Tian, Hongxiao Wu, Yameng Mu, Bingqing Ren, Ling Lin, Zhihai Chen

**Affiliations:** 1 National Key Laboratory of Intelligent Tracking and Forecasting for Infectious Diseases, Beijing Ditan Hospital, Capital Medical University, Beijing, China; 2 Department of Infectious Diseases, Yantai Qishan Hospital for Infectious Disease, Yantai, China

**Keywords:** cohort-based assessment, diagnostic stratification, invasive pulmonary aspergillosis, meta-analysis, severe fever with thrombocytopenia syndrome, SFTS-associated pulmonary aspergillosis

## Abstract

**Background:**

This study aimed to develop and assess a meta-analysis-informed diagnostic stratification tool for invasive pulmonary aspergillosis (IPA) in patients with severe fever with thrombocytopenia syndrome (SFTS).

**Methods:**

A systematic review and meta-analysis were conducted to identify bedside clinical factors associated with IPA/SFTS-associated pulmonary aspergillosis (SAPA) in patients with SFTS. Because individual participant data were unavailable, pooled study-level odds ratios were translated into a pragmatic three-variable diagnostic stratification score rather than a conventional individual-level prediction model. The tool was assessed in a separate single-center retrospective cohort of 220 patients with SFTS, among whom 63 (28.6%) met the clinically ascertained IPA/SAPA endpoint.

**Results:**

Six studies involving 2,342 patients with SFTS, including 407 patients with IPA/SAPA, were included in the quantitative meta-analysis. In the primary random-effects analyses, neurological symptoms showed the clearest pooled association with increased IPA/SAPA risk, whereas ICU admission and corticosteroid use showed positive but less precise pooled directions of effect. The final three-variable score assigned 9 points for neurological symptoms, 9 points for ICU admission, and 6 points for corticosteroid use, yielding a total score range of 0–24 points. In the single-center cohort-based assessment, the diagnostic stratification tool showed encouraging discrimination, with an AUC of 0.910 (95% CI, 0.869–0.951) and a Brier score of 0.097 after cohort-based logistic recalibration. The observed IPA/SAPA incidence increased stepwise across low-, medium-, and high-risk groups: 3.7% (4/108), 22.8% (13/57), and 83.6% (46/55), respectively.

**Conclusion:**

This meta-analysis-informed diagnostic stratification tool showed encouraging cohort-based risk separation in a single-center retrospective assessment. It may help prioritize fungal diagnostic work-up and surveillance intensity in patients with SFTS and support more targeted diagnostic resource allocation and antifungal stewardship. However, it should not be used as a stand-alone basis for antifungal treatment decisions or as a broadly generalizable individual-level prediction model. External validation, local recalibration, and prospective multicenter evaluation are required before clinical implementation.

**Systematic Review Registration:**

https://www.crd.york.ac.uk/prospero/display_record.php?RecordID=5115813, identifier CRD420251115813.

## 1 Introduction

Severe fever with thrombocytopenia syndrome (SFTS) is an emerging, tick-borne, acute haemorrhagic fever caused by *Bandavirus dabieense* (Yun et al., 2020). First identified in China in 2009, SFTS has since been reported in East and Southeast Asia, posing a potential risk of global spread (Yu et al., 2011; Takahashi et al., 2014; Yun et al., 2014; Li et al., 2018; Tran et al., 2019). SFTS is characterized by fever, thrombocytopenia, leukopenia, and rapid progression to multiple organ dysfunction, with an average case fatality rate (CFR) ranging from 12% to 50% (Li et al., 2018). The World Health Organization (WHO) categorized SFTS as a disease requiring the most critical research focus in its 2017 assessment (Yang et al., 2022).

A key complication of SFTS is virus-induced immunosuppression, which predisposes patients to secondary infections such as invasive pulmonary aspergillosis (IPA), a lethal co-infection. Although exposure to Aspergillus spores is ubiquitous, invasive aspergillosis (IA) develops mainly in susceptible hosts with impaired antifungal immune defenses (Ledoux and Herbrecht, 2023). Studies have confirmed that patients with SFTS, in particular, exhibit both cellular and humoral immune dysfunction, including leukopenia and depletion of key immune effector cells, thereby creating a permissive environment for fungal invasion (Song et al., 2023). Emerging evidence suggests that early management of IPA reduces mortality in patients with SFTS (Seong et al., 2021; Huang et al., 2025).

Beyond its relevance as a complication of SFTS, aspergillosis should also be viewed within the broader public health challenge posed by pathogenic fungi. Pathogenic fungal diseases are increasingly recognized as an underappreciated threat to public health, partly because their burden is difficult to measure accurately and because diagnostic capacity and surveillance systems remain uneven across regions (Miao et al., 2026; Yuan et al., 2026). Recent global estimates suggest that severe fungal diseases and chronic pulmonary aspergillosis affect millions of people annually and contribute substantially to global mortality, underscoring that invasive fungal diseases are not only opportunistic complications in individual patients but also an important population-level health problem (Denning, 2024). In this context, Aspergillus is particularly relevant because it is a major invasive mold pathogen affecting vulnerable populations, and the WHO fungal priority pathogen framework has further emphasized the need to strengthen fungal surveillance, diagnostic capacity, antifungal resistance monitoring, and public health interventions (Fisher and Denning, 2023).

In the post-pandemic era, the clinical and public health importance of aspergillosis has become more prominent. Expanding populations with immune dysfunction, increased exposure to intensive-care interventions, broader use of corticosteroids and immunomodulatory therapies, and delayed recognition of fungal disease may all increase the risk of invasive aspergillosis in vulnerable patients (Fisher and Denning, 2023; Yuan et al., 2026). These challenges are especially relevant to SFTS, in which virus-associated immune dysregulation, leukopenia, critical illness, and frequent need for intensive supportive care may create a permissive environment for Aspergillus invasion. At the same time, diagnosis of IPA remains difficult because clinical manifestations are nonspecific and definitive confirmation often requires integration of radiological findings, fungal biomarkers, respiratory sampling, culture, and, when feasible, bronchoscopy-based evaluation (Patterson et al., 2016). Therefore, delayed diagnosis may lead to missed opportunities for timely antifungal intervention, whereas indiscriminate empiric antifungal use may increase drug exposure, toxicity, cost, and selection pressure for antifungal resistance.

In this context, a practical diagnostic stratification tool may have value beyond individual bedside prediction. By identifying patients with SFTS who are less likely or more likely to require intensified fungal work-up, such a tool may help clinicians allocate limited diagnostic resources more efficiently, prioritize fungal biomarker testing and chest imaging for higher-priority patients, and reduce unnecessary empiric antifungal exposure in lower-priority patients. This diagnostics-driven approach is also consistent with antifungal stewardship, in which earlier and more targeted diagnostic evaluation can support appropriate antifungal use, reduce prolonged empiric therapy, and improve resource utilization without replacing clinical judgment (Chakrabarti et al., 2022). Therefore, early diagnostic stratification for IPA/SAPA in SFTS has both clinical and public health relevance, particularly in high-burden or resource-limited settings where universal intensive fungal work-up may not be feasible.

Although several studies have reported clinical factors associated with IPA or SFTS-associated pulmonary aspergillosis in patients with SFTS, the available evidence remains fragmented, and a simple evidence-based bedside tool for early diagnostic stratification is still lacking. Therefore, this study aimed to develop a meta-analysis-informed diagnostic stratification tool for IPA/SAPA in patients with SFTS and to assess its cohort-based performance in a separate single-center retrospective cohort. The intended role of this tool is to support diagnostic stratification and prioritization of fungal diagnostic work-up, including fungal biomarker testing, repeat chest imaging, and bronchoscopy/BAL when clinically appropriate, rather than to serve as a stand-alone treatment algorithm, an automatic trigger for antifungal therapy, or a fully validated individual-level prediction model.

## 2 Methods

This study was approved by the Ethics Committee of Beijing Ditan Hospital, Capital Medical University (Approval No. DTEC-KY2022-022-01) and conducted in compliance with the principles outlined in the Declaration of Helsinki. The requirement for written informed consent from each patient was waived due to the retrospective nature of the study. The registration of this systematic review and meta-analysis protocol was completed in PROSPERO (CRD420251115813).

### 2.1 Literature screening and study selection

A systematic search of PubMed, Web of Science, Embase, and the Cochrane Library was conducted for studies published up to May 5, 2025. The search employed a combination of terms, including “severe fever with thrombocytopenia syndrome,” “SFTS,” “invasive pulmonary aspergillosis,” “IPA,” and “risk factors.” Two investigators independently screened titles and abstracts, followed by full-text review of potentially eligible studies. Disagreements were resolved by discussion and, when necessary, consultation with a third reviewer. Original studies were considered eligible if they included laboratory-confirmed SFTS patients, directly evaluated risk factors for IPA or SAPA, and provided extractable effect estimates or comparable data for quantitative synthesis. Studies focusing primarily on broader pulmonary infection or invasive pulmonary fungal infection without directly evaluating IPA/SAPA as the primary outcome of interest were excluded from the primary quantitative meta-analysis. Detailed database-specific search strategies, additional search procedures, and eligibility criteria are provided in the Supplementary Material.

### 2.2 Data extraction

Two reviewers independently extracted data from each eligible study using a standardized form. Extracted information included the first author, year of publication, country or region, study design, sample size, number of IPA events, patient demographics (e.g., age, sex), reported risk factors, adjusted or unadjusted odds ratios (ORs) or risk ratios (RRs), and corresponding 95% confidence intervals (CIs). For bedside candidate predictors that were repeatedly reported across the included studies and had extractable effect estimates, reported odds ratios were extracted whenever available. These predictors included age, corticosteroid use, diabetes, ICU admission, and neurological symptoms. When both adjusted and unadjusted estimates were available for the same predictor, adjusted estimates were preferentially extracted; unadjusted estimates were used only when adjusted estimates were unavailable or when the adjusted model did not include the corresponding candidate predictor. When necessary, effect directions were harmonized so that odds ratios greater than 1 consistently indicated a higher risk of IPA/SAPA. Additional study-level methodological and clinical details are provided in **Supplementary Table 3**, and study-level effect estimates used in the pooled analyses are presented in **Supplementary Table 5**. Definitions of IPA/SAPA diagnostic frameworks across the included studies and the operational definitions of these five bedside candidate predictors are summarized in **Supplementary Table 6** and **S7**. Because fully uniform operational definitions were not available across all studies, study-specific variables were harmonized conservatively to these five bedside candidate predictors. As summarized in **Supplementary Table 7**, neurological symptoms encompassed article-reported central nervous system symptoms, tremor or related neurologic manifestations, or encephalopathy; corticosteroid use referred to article-reported systemic corticosteroid or glucocorticoid exposure during hospitalization, although dose, duration, and route were not uniformly reported; ICU admission included article-reported ICU admission or ICU transfer during hospitalization; diabetes included article-reported diabetes history or uncontrolled diabetes; and age was retained as reported in the source articles, although scaling was not fully equivalent across studies.

### 2.3 Single-center retrospective cohort for cohort-based assessment

A total of 261 patients diagnosed with SFTS at Yantai Qishan Hospital between January 2022 and December 2024 were initially enrolled as a separate single-center retrospective cohort for cohort-based performance assessment. The diagnosis of SFTS was confirmed if at least one of the following criteria was met: (a) isolation of the virus from patient samples; (b) detection of SFTS virus RNA in serum; or (c) a ≥4-fold increase in specific antibody titers between acute and convalescent paired serum samples. The exclusion criteria were as follows: patients with pre-existing immunodeficiency disorders, autoimmune diseases, malignancies, or other concurrent invasive fungal infections; those with missing critical laboratory results or clinical documentation; those who were pregnant or died within 24 h of admission; those who had received systemic antifungals for more than 48 h before admission; and those with pre-existing unresolved IPA before SFTS virus infection. After 41 patients were excluded, 220 were included in the final analysis. IPA ascertainment in the assessment cohort was based on retrospective review of clinical, radiologic, and mycological data documented in the medical records and interpreted using an EORTC/MSGERC-based framework adapted for this SFTS cohort. Because SFTS-associated immune dysfunction is not fully captured by classical EORTC/MSGERC host-factor definitions, this framework was used to structure radiologic and mycological evidence rather than to imply strict applicability of all host-factor criteria. Chest computed tomography findings were reviewed together with available fungal microbiological evidence, including serum galactomannan testing, bronchoscopy and bronchoalveolar lavage-based evaluation when available, respiratory specimen culture, blood culture, and occasional supportive NGS results. For research transparency, cases in the assessment cohort were further categorized as proven IPA, probable IPA, suspected only/empirically treated, or not classifiable as IPA according to the available radiologic and mycological evidence. Clinical suspicion or empiric/pre-emptive antifungal treatment alone was not considered sufficient for research classification of IPA unless accompanied by compatible radiologic findings and the required mycological or microbiological evidence. A summary of the final research classification and key diagnostic evidence is provided in **Supplementary Table 9**.

### 2.4 Meta-analysis

Among the risk factors reported in the eligible studies, five bedside candidate predictors had extractable effect estimates suitable for quantitative synthesis: age, corticosteroid use, diabetes, ICU admission, and neurological symptoms. Because clinical and methodological heterogeneity across studies was anticipated, random-effects models were used as the primary analytic approach. Between-study variance was estimated using restricted maximum likelihood (REML), and Hartung–Knapp adjustment was applied for random-effects confidence intervals. For sensitivity analysis, common-effect models were also fitted. Because only a small number of studies were available for each predictor and differences in study populations, IPA/SAPA diagnostic frameworks, and predictor operationalization were anticipated across studies, random-effects estimates were used for primary inference, whereas common-effect models were retained only as sensitivity analyses.

For consistency, pooled analyses were conducted on log-transformed odds ratios recalculated from the reported odds ratios and 95% confidence intervals using natural logarithms. Effect directions were harmonized so that an odds ratio greater than 1 indicated increased IPA/SAPA risk. Statistical heterogeneity was assessed using the I² statistic, τ², and Cochran’s Q test. Leave-one-out analyses were performed when at least three studies were available for a given predictor. Funnel plots were generated only when feasible and were interpreted cautiously because of the small number of studies per predictor.

A stricter-definition sensitivity analysis excluding studies with broader SAPA definitions was planned to assess the influence of outcome-definition heterogeneity. However, after exclusion of these studies, fewer than two studies remained for each predictor, and formal pooled strict-definition sensitivity analyses were therefore not estimable. Outcome-definition heterogeneity was instead addressed by using random-effects models for primary inference and by documenting study-level diagnostic frameworks in **Supplementary Table 6**. Predictor-definition heterogeneity was addressed by conservative study-level harmonization and cautious interpretation of pooled estimates in light of **Supplementary Table 7**. Results of the common-effect sensitivity analyses are provided in **Supplementary Table 8**.

### 2.5 Construction of the diagnostic stratification score

Because individual participant data from the studies included in the meta-analysis were not available, the diagnostic stratification score was not developed by fitting a *de novo* multivariable individual-level prediction model across pooled patients. Instead, we constructed a meta-analysis-informed bedside diagnostic stratification score using pooled study-level effect estimates from the primary random-effects meta-analysis. For each bedside candidate predictor included in the pooled analysis, the pooled odds ratio was converted to the natural-log scale and considered as an aggregate evidence-based weight.

Because the available evidence was derived from a small number of studies and from study-level rather than individual-level data, point assignment was treated as a pragmatic weighting scheme rather than as individual-level regression coefficients. The retained predictors were selected according to the consistency, clinical interpretability, and bedside availability of the pooled associations. Neurological symptoms, ICU admission, and corticosteroid use were retained in the primary diagnostic stratification score because they represented clinically accessible variables with positive pooled directions of effect and plausible relevance to IPA/SAPA risk in SFTS.

To avoid assigning overly extreme weights, we applied a conservative coefficient-stabilization and point-scaling procedure. A global shrinkage factor was calculated as omega = max(0, 1 − p/n_events), where p = 3 was the number of retained predictors and n_events = 63 was the number of clinically ascertained IPA/SAPA events in the assessment cohort. Variable-specific shrinkage factors were then calculated using normalized precision weights derived from the pooled meta-analytic estimates. The shrunken coefficients were linearly rescaled so that the sum of integer points equaled 24 points. This process yielded 9 points for neurological symptoms, 9 points for ICU admission, and 6 points for corticosteroid use, with a total score range of 0–24 points.

Probability calibration was performed only during the cohort-based assessment and was not used to change the assigned point values. The step-by-step derivation of the final point values is provided in **Supplementary Table 10**.

### 2.6 Cohort-based performance assessment

The meta-analysis-informed diagnostic stratification tool was assessed in a separate single-center retrospective cohort of 220 patients with SFTS as an initial cohort-based performance evaluation rather than as independent external validation. The primary assessment used the clinically ascertained IPA/SAPA endpoint recorded in the retrospective cohort, which included 63 cases (28.6%). For transparency, these 63 clinically ascertained cases were further separated into 51 probable IPA cases and 12 suspected only/empirically treated cases according to the final research classification, with 157 patients classified as not classifiable as IPA. A summary of the final research classification and key diagnostic evidence is provided in **Supplementary Table 9**.

Discrimination was evaluated using the area under the receiver operating characteristic curve (AUC), with 95% CIs estimated using DeLong’s method. Apparent calibration after cohort-based logistic recalibration was examined using calibration plots and the Brier score. Decision curve analysis was used to explore the potential utility of the tool for risk-stratified fungal diagnostic escalation, such as fungal biomarker testing, repeat chest imaging, and consideration of bronchoscopy/BAL, rather than to define a treatment-triggering threshold or to support automatic antifungal treatment initiation.

To evaluate robustness to diagnostic-classification uncertainty, an additional sensitivity analysis was performed using a stricter probable-IPA endpoint. In this analysis, the 12 patients categorized as suspected only/empirically treated were reclassified as non-IPA, and only proven or probable IPA was considered an event. The same point allocation and predefined risk-group thresholds were retained. All analyses were performed in R (version 4.3.0) using the meta, pROC, boot, and rmda packages.

## 3 Results

### 3.1 Literature selection and study characteristics

**Figure 1:**
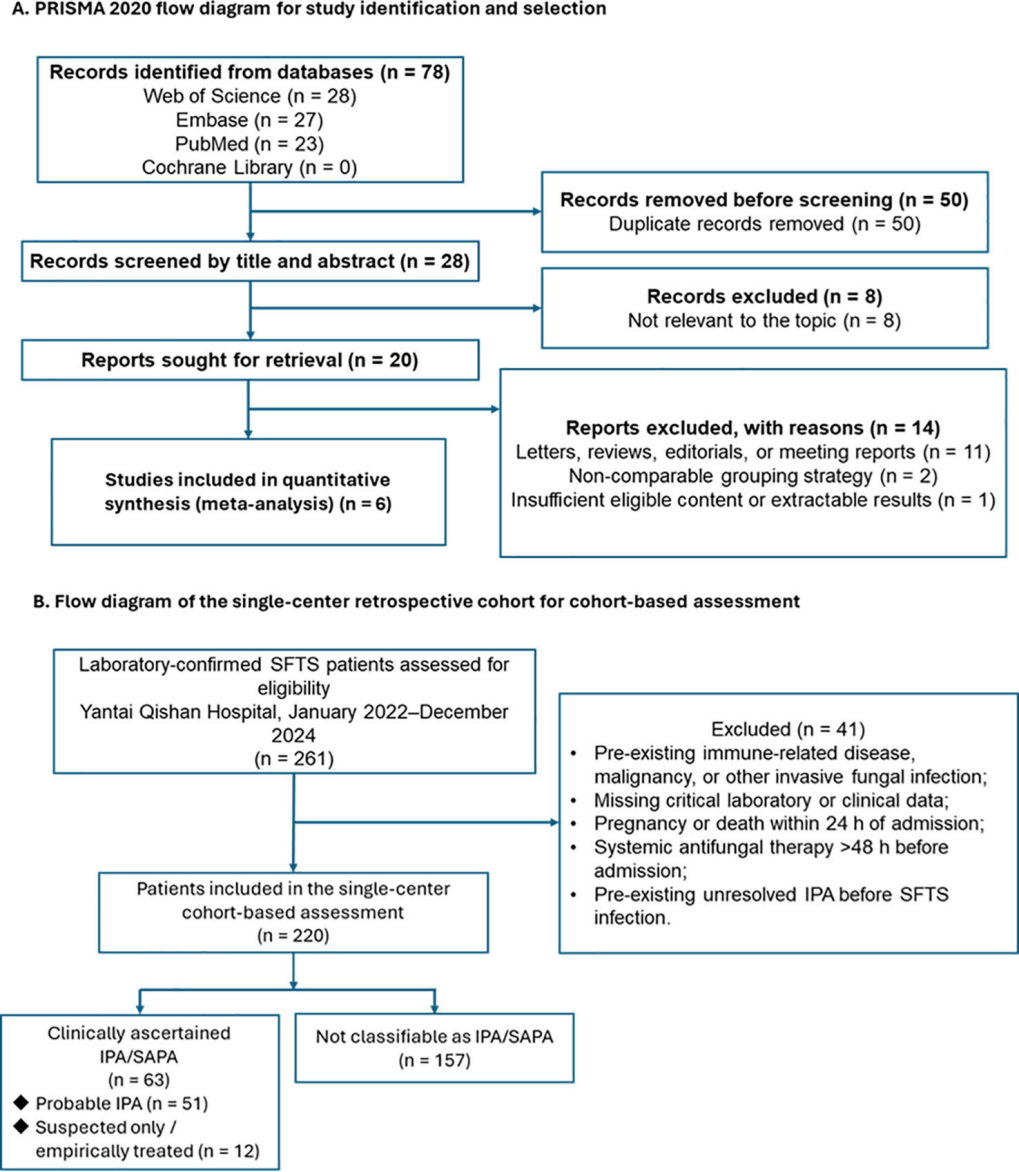
Flow diagrams of study selection and cohort construction. **(A)** PRISMA 2020 flow diagram showing identification, screening, eligibility assessment, and inclusion of studies for the systematic review and meta-analysis. **(B)** Flow diagram of patient enrollment and exclusion in the single-center retrospective cohort for cohort-based assessment. Clinically ascertained IPA/SAPA included probable IPA and suspected only/empirically treated cases; detailed diagnostic evidence is provided in **Supplementary Table 9**. IPA, invasive pulmonary aspergillosis; SAPA, SFTS-associated pulmonary aspergillosis; SFTS, severe fever with thrombocytopenia syndrome.

A total of 78 records were identified through systematic searches of Web of Science (n = 28), Embase (n = 27), PubMed (n = 23), and the Cochrane Library (n = 0). After removal of 50 duplicate records, 28 records underwent title and abstract screening, of which eight were excluded as not relevant to the review question. The remaining 20 reports were sought for retrieval and assessed for eligibility. Fourteen reports were excluded for the following reasons: letters, reviews, editorials, or meeting reports (n = 11), non-comparable grouping strategies (n = 2), and insufficient eligible content or extractable results (n = 1). Ultimately, six studies met the inclusion criteria and were included in the quantitative meta-analysis (**Figure 1A**). Detailed characteristics of the included studies are presented in **Supplementary Table 1**, quality assessment results in **Supplementary Table 2**, additional methodological and clinical details in **Supplementary Table 3**, and study-level effect estimates used in the pooled analyses in **Supplementary Table 5**.

The six eligible studies included a total of 2,342 patients with SFTS, including 407 patients with IPA/SAPA. The candidate predictors evaluated across studies included age, corticosteroid use, diabetes, ICU admission, and neurological symptoms. However, the availability of study-level data differed by predictor, and both exposure definitions and diagnostic frameworks varied across studies. These cross-study differences are summarized in **Supplementary Table S6** and **S7**.

### 3.2 Characteristics of the single-center retrospective cohort for cohort-based assessment

A total of 220 individuals diagnosed with SFTS were enrolled in the single-center retrospective cohort for cohort-based assessment, with the selection workflow and corresponding results depicted in **Figure 1B**. The median age and hospitalization duration of the overall cohort were 68 years and 9 days, respectively, and 51.8% were female. Based on the clinically applied IPA ascertainment framework used in this retrospective cohort, 63 patients met the clinically ascertained IPA/SAPA endpoint, whereas 157 were not classifiable as IPA. For research transparency, final evidence-based classification further identified 0 patients as proven IPA, 51 (23.2%) as probable IPA, 12 (5.5%) as suspected only/empirically treated, and 157 (71.4%) as not classifiable as IPA (**Supplementary Table 9**). Among the 51 probable IPA cases, compatible chest CT findings were present in all cases, serum galactomannan was positive in 40 (78.4%), bronchoscopy was performed in 47 (92.2%), respiratory specimen culture was positive in 41 (80.4%), and supportive NGS results were available in 2 (3.9%). No cases had histopathologic or sterile-site evidence consistent with proven invasive disease. Compared with the non-IPA group, patients with clinically ascertained IPA had longer hospital stays and higher frequencies of petechiae, cough, dyspnea, and tremor. Differences were also observed in platelet count, inflammatory markers, tissue-injury markers, renal function, electrolytes, and coagulation parameters, as detailed in **Supplementary Table 4**.

### 3.3 Meta-analysis of predictors and construction of the diagnostic stratification score

**Figure 2:**
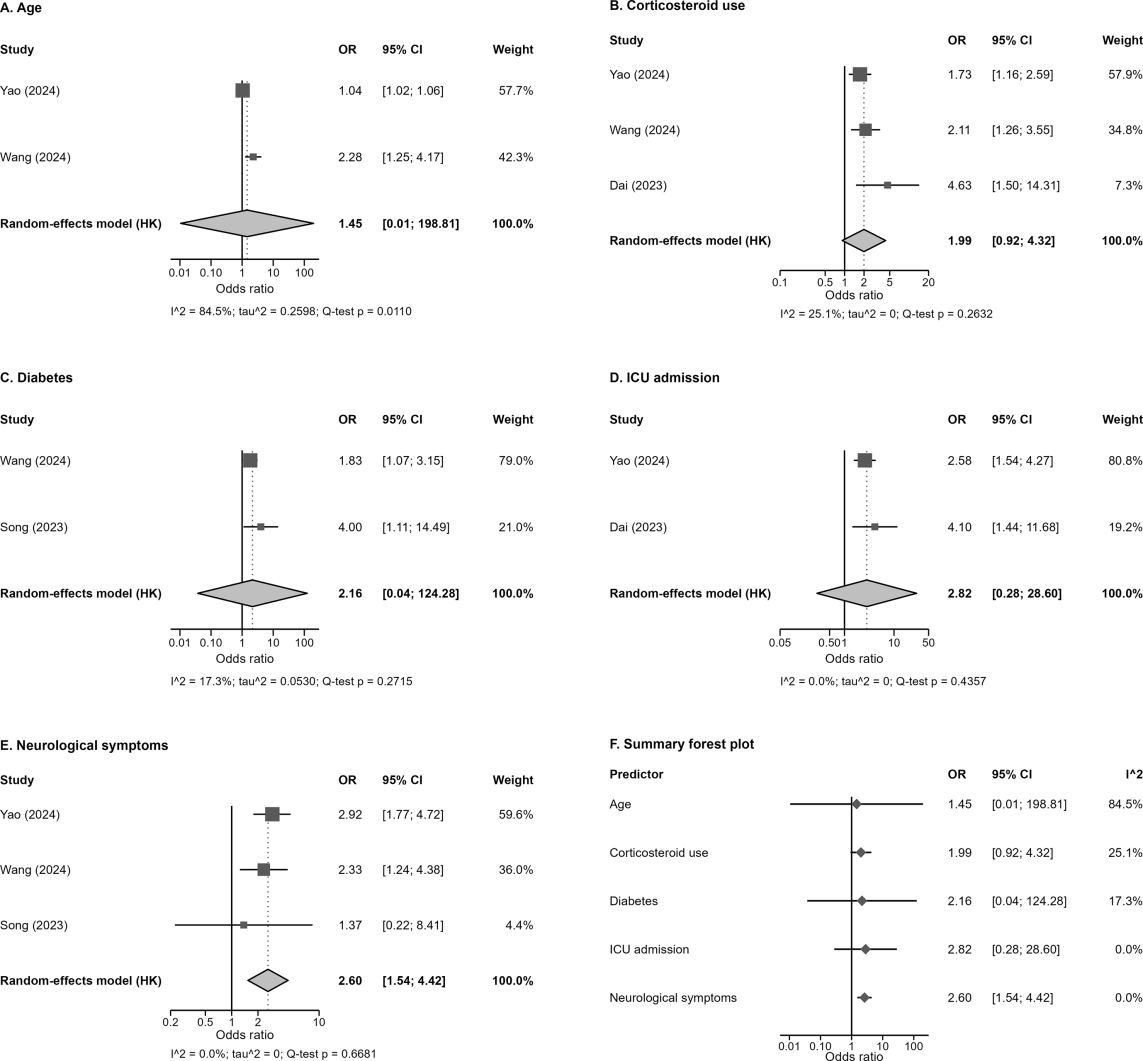
Predictor-specific forest plots from the primary random-effects meta-analysis and summary forest plot of pooled estimates. **(A)** Age. **(B)** Corticosteroid use. **(C)** Diabetes. **(D)** ICU admission. **(E)** Neurological symptoms. **(F)** Summary forest plot of the primary random-effects pooled estimates for the five bedside candidate predictors. OR, odds ratio; CI, confidence interval. Random-effects estimates were generated using restricted maximum likelihood (REML) with Hartung–Knapp adjustment.

Pooled results for the five bedside candidate predictors included in the quantitative synthesis are summarized in **Table 1**, and the corresponding study-level effect estimates are provided in **Supplementary Table 5**. Under the primary random-effects model with REML and Hartung–Knapp adjustment, neurological symptoms showed the clearest pooled association with increased IPA/SAPA risk (OR 2.60, 95% CI 1.54–4.42). ICU admission also showed a positive pooled point estimate (OR 2.82, 95% CI 0.28–28.60), but the confidence interval was wide because only two studies contributed data and Hartung–Knapp adjustment was applied. Corticosteroid use showed a positive but less precise association (OR 1.99, 95% CI 0.92–4.32) (**Figure 2A–F**). Diabetes also showed a positive pooled direction of effect (OR 2.16, 95% CI 0.04–124.28), but interpretation remained cautious because of limited study numbers and between-study differences in exposure definitions. Age was not emphasized in the diagnostic stratification score because the contributing studies used non-equivalent scaling approaches, limiting direct comparability of the pooled estimate.

**Table 1:** Primary random-effects meta-analysis of five candidate bedside predictors for IPA/SAPA in patients with SFTS.

Predictor	No. of studies	Random-effects OR (95% CI)	P value	I² (%)	Q-test P value
Age	2	1.45 (0.01–198.81)	0.514	84.5	0.011
Corticosteroid use	3	1.99 (0.92–4.32)	0.062	25.1	0.263
Diabetes	2	2.16 (0.04–124.28)	0.250	17.3	0.271
ICU admission	2	2.82 (0.28–28.60)	0.111	0.0	0.436
Neurological symptoms	3	2.60 (1.54–4.42)	0.016	0.0	0.668

IPA, invasive pulmonary aspergillosis; SAPA, SFTS-associated pulmonary aspergillosis; OR, odds ratio; CI, confidence interval. Random-effects estimates were generated using restricted maximum likelihood (REML) with Hartung–Knapp adjustment. Age should be interpreted cautiously because the contributing studies used non-equivalent age scaling approaches. Diabetes should also be interpreted cautiously because only two studies contributed data and exposure definitions were not fully uniform

Regarding statistical heterogeneity, corticosteroid use showed low-to-moderate heterogeneity (I² = 25.1%), whereas ICU admission and neurological symptoms showed no observed statistical heterogeneity (both I² = 0%). However, the absence of observed statistical heterogeneity should be interpreted cautiously because only a small number of studies contributed to each predictor-specific analysis. Age showed substantial heterogeneity (I² = 84.5%), likely reflecting non-equivalent scaling approaches across studies rather than a uniform bedside effect estimate. These findings support interpretation of the pooled estimates in the context of cross-study differences in outcome frameworks and predictor operationalization.

Common-effect sensitivity analyses showed effect directions consistent with the primary random-effects analyses for all five bedside candidate predictors, although statistical significance was more pronounced under the common-effect sensitivity approach for some predictors (**Supplementary Table 8**). Because heterogeneity in outcome definitions and predictor operationalization was anticipated across studies, random-effects estimates were interpreted as the primary results. Regarding outcome-definition heterogeneity, four included studies were classified as IPA-focused and two as broader SAPA-oriented studies (**Supplementary Table 6**). To assess the influence of outcome-definition heterogeneity, a stricter-definition sensitivity analysis excluding the broader SAPA-oriented studies was considered; however, formal pooled predictor-specific analyses were not estimable because fewer than two studies remained for each predictor after exclusion. Regarding predictor-definition heterogeneity, neurological symptoms were harmonized from article-specific terms such as central nervous system symptoms, tremor or related neurologic manifestations, and encephalopathy; corticosteroid use generally referred to systemic corticosteroid or glucocorticoid exposure during hospitalization, although dose, duration, and route were inconsistently reported; ICU-related exposure included ICU admission or ICU transfer during hospitalization; and diabetes ranged from general diabetes history to uncontrolled diabetes across studies (**Supplementary Table 7**). 

**Figure 3:**
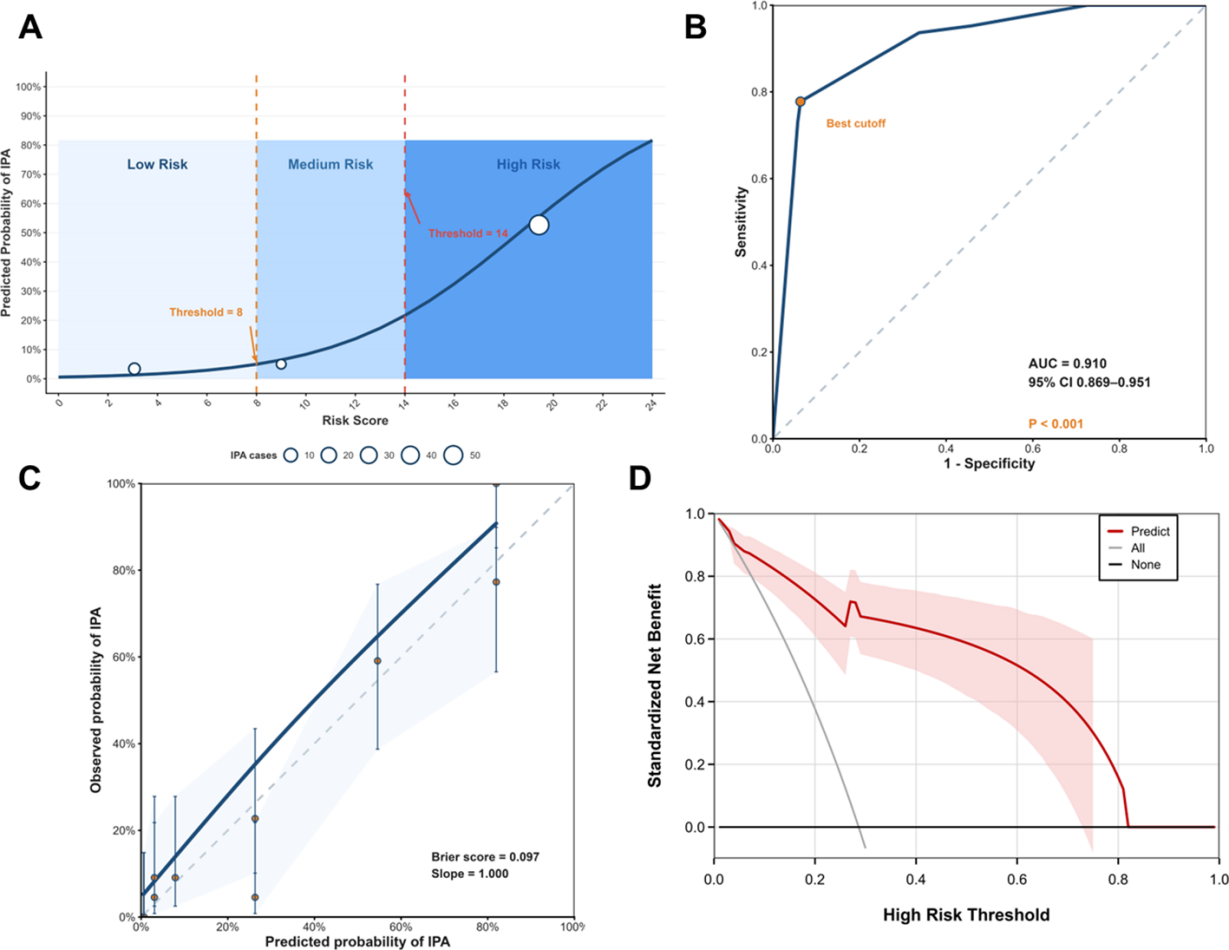
Score-risk relationship and cohort-based performance assessment of the meta-analysis-informed diagnostic stratification tool. **(A)** Score-stratified risk curve illustrating the relationship between the summed three-component score (range 0–24 points) and the cohort-recalibrated predicted probability of IPA/SAPA. This curve is intended as a cohort-based visualization and should not be interpreted as an externally calibrated individual risk-prediction function. **(B)** Receiver operating characteristic curve with area under the curve (AUC). **(C)** Calibration plot comparing predicted and observed probabilities after cohort-based logistic recalibration. **(D)** Decision curve analysis evaluating potential utility of the tool for risk-stratified fungal diagnostic escalation, rather than defining a treatment-triggering threshold or supporting automatic antifungal treatment initiation. AUC, area under the receiver operating characteristic curve; CI, confidence interval; IPA, invasive pulmonary aspergillosis; SAPA, SFTS-associated pulmonary aspergillosis.

The pooled evidence was then translated into a meta-analysis-informed three-variable diagnostic stratification score incorporating neurological symptoms, ICU admission, and corticosteroid use. After coefficient stabilization and linear point scaling, 9 points were assigned for neurological symptoms, 9 points for ICU admission, and 6 points for corticosteroid use. The total score was calculated as the sum of these three component scores, yielding a range of 0–24 points. The relationship between the total score and cohort-recalibrated predicted IPA/SAPA probability is shown in **Figure 3A**, and the detailed score-construction steps are summarized in **Supplementary Table 10**. Because the point values were derived from study-level pooled estimates rather than individual participant data, this score should be interpreted as a pragmatic evidence-informed diagnostic stratification tool rather than a fully specified individual-level prediction equation.

### 3.4 Cohort-based performance assessment

The diagnostic stratification tool showed encouraging discrimination in the single-center cohort-based assessment, with an AUC of 0.910 (95% CI 0.869–0.951) (**Figure 3B**). At the Youden-derived probability threshold of 0.275, sensitivity was 0.778, specificity was 0.936, PPV was 0.831, NPV was 0.913, and overall accuracy was 0.891 (**Table 2**).

**Table 2:** Cohort-based performance assessment of the meta-analysis-informed diagnostic stratification tool.

A. Overall cohort-based performance					
Measure			Value		
No. of patients			220		
IPA/SAPA events, n (%)			63 (28.6)		
Point allocation			Neurological symptoms = 9; ICU admission = 9; corticosteroid use = 6		
Total score range			0–24		
AUC (95% CI)			0.910 (0.869–0.951)		
Youden-derived probability threshold			0.275		
Sensitivity			0.778		
Specificity			0.936		
PPV			0.831		
NPV			0.913		
Accuracy			0.891.		
Brier score			0.097		
B. Risk-stratum distribution and observed IPA/SAPA incidence					
Risk stratum	Score range	No. of patients		IPA/SAPA events	Observed incidence, % (95% CI)
Low risk	0–9	108		4	3.7 (1.4–9.1)
Medium risk	15	57		13	22.8 (13.8–35.2)
High risk	24	55		46	83.6 (71.7–91.1)

AUC, area under the receiver operating characteristic curve; CI, confidence interval; IPA, invasive pulmonary aspergillosis; NPV, negative predictive value; PPV, positive predictive value; SAPA, SFTS-associated pulmonary aspergillosis.

The updated three-variable score assigned 9 points for neurological symptoms, 9 points for ICU admission, and 6 points for corticosteroid use. Predicted probabilities were obtained through logistic recalibration of the study-level meta-analysis-informed linear predictor in the assessment cohort. The Youden-derived probability threshold is reported only for descriptive cohort-based classification performance and should not be interpreted as a treatment-triggering threshold or as support for automatic antifungal treatment initiation.

Risk strata were defined by the total-score thresholds used in the cohort-based assessment: low risk, 0–9 points; medium risk, 15 points; and high risk, 24 points. Because the score is composed of three binary variables, only discrete total-score values were observed in the cohort.

These findings represent cohort-based assessment rather than external validation and should not be interpreted as evidence that the diagnostic stratification tool is ready for routine clinical implementation without external validation, local recalibration, and prospective multicenter evaluation. These cohort-based performance estimates are intended to describe diagnostic stratification and should not be used to define antifungal.

After logistic recalibration of the meta-analysis-informed linear predictor in the assessment cohort, the tool showed favorable apparent calibration, with a Brier score of 0.097 (**Figure 3C**). Because this recalibration was performed within the same cohort, these calibration findings should be interpreted as cohort-based apparent calibration rather than external calibration.

Exploratory decision curve analysis suggested that use of the tool to guide risk-stratified fungal diagnostic escalation provided higher standardized net benefit than strategies of diagnostic escalation for all patients or no diagnostic escalation across much of the examined threshold-probability range (**Figure 3D**). This analysis was intended to evaluate diagnostic escalation, such as fungal biomarker testing, repeat chest CT, or consideration of bronchoscopy and BAL, and should not be interpreted as defining a treatment-triggering threshold or supporting automatic antifungal treatment initiation.

In the diagnostic-classification sensitivity analysis, the 12 patients categorized as suspected only/empirically treated were reclassified as non-IPA, leaving 51 probable IPA cases as events and 169 patients as non-events. Under this stricter endpoint definition, discrimination was preserved, with an AUC of 0.933 (95% CI 0.899–0.967). Using the ROC-derived cutoff probability of 0.322, sensitivity was 0.882, specificity was 0.917, PPV was 0.763, NPV was 0.963, accuracy was 0.909, and the Brier score was 0.106. These findings suggest that the cohort-based performance was not driven solely by clinically suspected or empirically treated cases with less certain IPA classification (**Supplementary Table 11**; **Supplementary Figure 1**).

### 3.5 Diagnostic risk stratification in the assessment cohort

**Figure 4:**
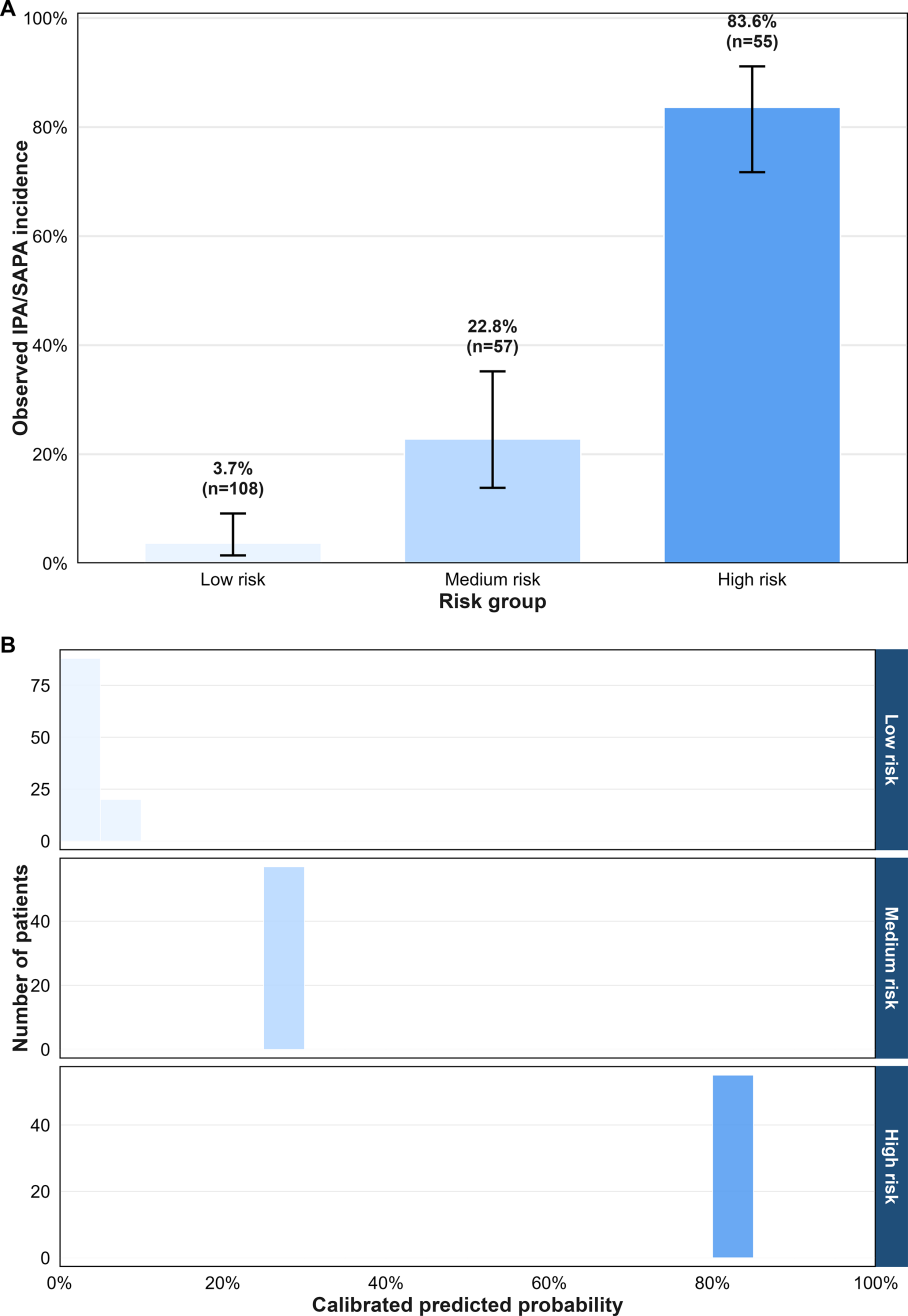
Diagnostic risk stratification based on the meta-analysis-informed three-variable score. **(A)** Observed IPA/SAPA incidence across low-, medium-, and high-risk groups. **(B)** Distribution of cohort-recalibrated predicted probabilities across low-, medium-, and high-risk groups. Risk groups were defined as low risk (0 -9 points), medium risk (10 -14 points), high risk (15 -24 points). These groups are intended to support diagnostic prioritization, risk-stratified surveillance, and fungal diagnostic escalation, rather than to serve as treatment-decision categories or automatic triggers for antifungal therapy. low risk (0–9 points), medium risk (10–14 points), high risk (15–24 points).

Because the diagnostic stratification score was composed of three binary variables, only discrete score values were observed in the assessment cohort. Patients were classified into low-, medium-, and high-risk strata corresponding to total scores of 0–9, 15, and 24 points, respectively. The observed IPA/SAPA incidence increased stepwise across these strata: 3.7% (4/108; 95% CI 1.4%–9.1%) in the low-risk group, 22.8% (13/57; 95% CI 13.8%–35.2%) in the medium-risk group, and 83.6% (46/55; 95% CI 71.7%–91.1%) in the high-risk group (**Figure 4**; **Table 2**).

When the stricter probable-IPA endpoint was applied, the diagnostic stratification pattern was preserved. The observed probable IPA rates were 0.9% (1/108; 95% CI 0.2%–5.1%) in the low-risk group, 12.3% (7/57; 95% CI 6.1%–23.2%) in the medium-risk group, and 78.2% (43/55; 95% CI 65.6%–87.1%) in the high-risk group. Thus, the predefined risk categories showed clear separation even under a more conservative diagnostic classification (**Supplementary Table 11**; **Supplementary Figure 1**).

## 4 Discussion

In this study, we constructed a meta-analysis-informed diagnostic stratification tool for IPA/SAPA in patients with SFTS and assessed its performance in a separate single-center retrospective cohort. Among the five bedside candidate predictors included in the quantitative synthesis, neurological symptoms showed the clearest pooled association with IPA/SAPA risk, whereas ICU admission and corticosteroid use showed positive but less precise pooled directions of effect. These findings were translated into a pragmatic three-variable diagnostic stratification score incorporating neurological symptoms, ICU admission, and corticosteroid use. In the cohort-based assessment, the tool showed encouraging discrimination and clear separation of observed IPA/SAPA incidence across predefined diagnostic risk strata, but these findings should be interpreted as preliminary because the tool was derived from study-level aggregate estimates and assessed in a single-center cohort with a relatively high IPA/SAPA prevalence. Accordingly, the observed discrimination and risk separation should be interpreted as supportive evidence for diagnostic stratification in this cohort, not as proof of a broadly transportable individual-level prediction model.

Our findings are consistent with previous studies suggesting that immune dysfunction and critical illness may predispose patients with SFTS to fungal co-infections (Romani, 2011; Hu et al., 2021). SFTS is characterized by virus-associated immune dysregulation, including leukopenia and impaired cellular immune responses, which may create a permissive host environment for secondary fungal invasion. Advanced age may further contribute to IPA susceptibility through immunosenescence and reduced physiologic reserve (Yao et al., 2024). Diabetes may also increase vulnerability to invasive fungal infection through chronic hyperglycemia-related impairment of neutrophil chemotaxis and phagocytosis, as well as microvascular dysfunction that affects local immune defense (Trof et al., 2007; Song et al., 2023). Although age and diabetes remain clinically relevant candidate factors, they were not emphasized in the current primary diagnostic stratification score because the pooled evidence for these variables was less directly transferable to a parsimonious bedside diagnostic tool.

Neurological involvement showed one of the most consistent associations with IPA/SAPA risk in the quantitative synthesis. Previous studies have shown that neurological symptoms are frequently associated with severe SFTS and may indicate a higher risk of adverse outcomes (Gai et al., 2012; Song et al., 2022). In the context of IPA risk, neurological involvement may reflect a more severe systemic disease state, potentially accompanied by higher viral burden, immune dysregulation, and multiorgan dysfunction. Neurological impairment may also compromise airway protection, weaken effective cough, increase aspiration risk, and delay recognition of evolving respiratory deterioration. Studies of neurological disease-associated pneumonia and SFTS-associated IPA further suggest that neurological dysfunction and critical illness may be linked to altered respiratory microbiology and increased pulmonary infection risk (Zhao et al., 2021; Wang et al., 2024). Together, these factors could plausibly create conditions that favor Aspergillus colonization or invasion in vulnerable patients. Therefore, neurological signs in patients with SFTS should prompt closer clinical reassessment and, when accompanied by respiratory deterioration or compatible imaging findings, earlier consideration of fungal diagnostic work-up.

This study also highlights the combined impact of corticosteroid use and ICU admission on the risk of IPA in patients with SFTS. Although corticosteroids are often prescribed to control excessive inflammation or cytokine storms in severe cases, they may weaken innate antifungal immunity by suppressing alveolar macrophage function and reducing the ability of neutrophils to destroy *Aspergillus* hyphae (Balloy et al., 2005; Wang et al., 2024). This immune suppression compromises key defenses against fungal invasion. Meanwhile, ICU admission, while essential for providing advanced respiratory and circulatory support, exposes patients to additional risk factors, including invasive ventilation, broad-spectrum antibiotic use, and potential environmental exposure to fungal spores. When corticosteroid-induced immunosuppression coincides with the high-risk ICU setting, these factors may act synergistically, substantially increasing the chance of fungal colonization and progression to invasive disease. From a management perspective, these findings suggest that patients with SFTS receiving corticosteroids in the ICU may represent a subgroup at particularly high risk and may benefit from intensified surveillance. In such cases, closer surveillance with fungal biomarkers such as galactomannan, repeat chest imaging, and individualized diagnostic reassessment may facilitate earlier recognition of IPA. A balanced approach that maximizes the benefits of corticosteroid therapy and intensive care while minimizing infection risks is essential for improving outcomes in this vulnerable population.

From a clinical and public health perspective, the main value of this tool lies in diagnostic stratification and prioritization of fungal work-up, rather than in treatment decision-making. In real-world SFTS care, decisions about fungal work-up often must be made before definitive mycological evidence is available, particularly in patients with neurological deterioration, ICU transfer, or corticosteroid exposure. Fungal diagnostic resources such as serial galactomannan testing, repeat chest CT, bronchoscopy, and BAL are not always uniformly available or feasible for all patients. A simple bedside diagnostic stratification score may therefore help clinicians prioritize monitoring intensity and fungal diagnostic escalation for higher-priority patients, while supporting more conservative monitoring in low-priority patients. However, the cohort-recalibrated probabilities should not be interpreted as universally transportable individual risk estimates. This diagnostics-oriented approach may improve allocation of diagnostic resources, reduce unnecessary empiric antifungal exposure, and support diagnostics-driven antifungal stewardship.

For patients in the low-risk group (0–9 points), the observed IPA/SAPA incidence was low (3.7%), suggesting that the score alone does not support routine intensive fungal diagnostic work-up in the absence of other concerning clinical features. Standard clinical monitoring with reassessment according to the evolving disease course may be sufficient, and fungal biomarker testing or repeat chest imaging may be reserved for patients who subsequently develop new respiratory symptoms, worsening oxygenation, or compatible radiologic abnormalities.

For patients in the medium-risk group (15 points), the observed IPA/SAPA incidence was higher (22.8%), supporting closer surveillance and a lower threshold for diagnostic reassessment. In such patients, repeat clinical evaluation, serum fungal biomarker testing such as galactomannan and β-D-glucan where locally used, and repeat chest CT may be considered if respiratory symptoms worsen, fever remains unexplained, oxygenation deteriorates, or ICU transfer becomes likely. Bronchoscopy-based evaluation may also be considered when clinically feasible and when imaging findings raise concern for IPA.

For patients in the high-risk group (24 points), the observed IPA/SAPA incidence was markedly elevated (83.6%), suggesting that this subgroup may benefit from intensified surveillance, early multidisciplinary review, and a lower threshold for comprehensive fungal diagnostic work-up. In these patients, clinicians may reasonably consider early and repeated serum fungal biomarkers, repeat chest imaging, and bronchoscopy/BAL-based assessment when feasible, particularly when respiratory deterioration or new infiltrates are present. However, even in this high-risk group, the tool should not be used in isolation as an automatic trigger for prophylactic, pre-emptive, or therapeutic antifungal treatment. Antifungal treatment decisions should remain individualized and should integrate radiologic findings, microbiological evidence, overall disease severity, drug toxicity, and competing clinical considerations.

Overall, the score is best interpreted as a practical tool for diagnostic stratification, surveillance prioritization, and fungal diagnostic escalation. The resulting strata should not be interpreted as treatment-decision categories. The tool may help identify patients who warrant closer fungal monitoring and earlier diagnostic work-up, but it should complement rather than replace clinical judgment, microbiological testing, and imaging-based assessment.

Our study integrated evidence from multiple studies through meta-analysis and assessed the resulting diagnostic stratification score in a separate single-center retrospective cohort. This provides an initial cohort-based performance evaluation rather than true external validation. Accordingly, the present findings should not be interpreted as evidence of independent external validation or as support for broadly generalizable individual-level risk prediction. Rather, they should be viewed as supportive single-center evidence of diagnostic risk separation that requires confirmation, local recalibration, and prospective multicenter evaluation. Therefore, the AUC, apparent calibration, and observed risk-stratum probabilities reported here should be viewed as cohort-based performance indicators, not as evidence that the score is externally validated or ready for direct implementation across different clinical settings.

The coefficient-stabilization and point-scaling procedure was intended to avoid overly extreme point weights when translating pooled study-level associations into a bedside diagnostic stratification score. However, this procedure should not be interpreted as resolving the fundamental limitation of using aggregate study-level data. Because the score was not derived from individual participant data, the assigned point values may not fully reproduce individual-level predictor effects or predictor interactions. Therefore, the score should be interpreted as a pragmatic evidence-informed weighting scheme rather than as a conventional multivariable prediction model.

Cohort ascertainment reflected real-world SFTS practice, in which antifungal work-up intensity and treatment decisions may vary according to clinical severity, evolving imaging findings, availability of bronchoscopy or fungal biomarker testing, and physician concern for invasive fungal disease. In the final research classification, no cases met criteria for proven IPA, 51 were categorized as probable IPA, and 12 additional patients were classified as suspected only/empirically treated rather than proven or probable IPA. This distinction improves transparency and indicates that part of the clinically ascertained IPA/SAPA burden in this cohort may have been influenced by high clinical suspicion and early antifungal treatment in complex cases, rather than by uniformly confirmed invasive disease.

The diagnostic-classification sensitivity analysis showed that the diagnostic risk-separation pattern was preserved when the 12 suspected only/empirically treated cases were reclassified as non-IPA. Under this stricter probable-IPA endpoint, the AUC was 0.933, and the observed probable IPA rate increased from 0.9% in the low-risk group to 12.3% in the medium-risk group and 78.2% in the high-risk group. This finding supports the robustness of the diagnostic stratification pattern while also reinforcing that diagnostic uncertainty is an important limitation in retrospective real-world IPA ascertainment.

However, several limitations should be acknowledged. First, the score was constructed from pooled study-level effect estimates rather than from an individual participant data meta-analysis. Accordingly, the assigned predictor weights reflect aggregate between-study associations and may not fully reproduce individual-level relationships, introducing ecological bias and limiting individual-level interpretability. Second, the number of eligible studies was small, and only a limited number of studies contributed to each predictor-specific pooled estimate. Third, IPA/SAPA diagnostic frameworks and predictor definitions varied across studies. Although these differences were documented in **Supplementary Table 6** and **S7** and addressed through random-effects models and conservative harmonization, the pooled estimates should still be interpreted as synthesizing related but not fully identical outcome frameworks and clinical constructs. In addition, the planned stricter-definition sensitivity analysis excluding broader SAPA-oriented studies was not formally estimable because fewer than two studies remained for each predictor after exclusion. Fourth, the score was assessed only in a single-center retrospective cohort rather than in a true external validation dataset. Fifth, IPA ascertainment in the assessment cohort relied on retrospective real-world clinical, radiologic, and microbiological data, and bronchoscopy, bronchoalveolar lavage-based work-up, fungal biomarker testing, and other invasive procedures were not uniformly available in all patients. Therefore, some degree of diagnostic misclassification cannot be excluded. Finally, the relatively high clinically ascertained IPA/SAPA proportion in this cohort deserves particular caution. The observed prevalence of 28.6% was higher than that reported in some multicenter SFTS cohorts and may reflect center-specific case mix, referral patterns, diagnostic intensity, and early empiric antifungal treatment in clinically complex cases. Because PPV and observed risk-stratum probabilities are strongly influenced by baseline disease prevalence, the high-risk group incidence and positive predictive value observed in this cohort may not be directly transportable to centers with lower baseline IPA/SAPA risk. In such settings, the PPV would be expected to decrease, and local recalibration of baseline risk would be required before clinical use. Therefore, the present probability estimates should be regarded as cohort-recalibrated estimates rather than universally applicable individual risk probabilities. These limitations highlight the need for larger multicenter studies with standardized predictor and outcome definitions, external validation, local recalibration, and prospective evaluation before routine clinical implementation.

In summary, this meta-analysis-informed diagnostic stratification tool may provide a practical approach for early IPA/SAPA diagnostic stratification in patients with SFTS, particularly for prioritizing fungal surveillance and diagnostic escalation among higher-priority patients. It is not intended to replace clinical judgment, microbiological testing, imaging-based assessment, or individualized antifungal treatment decisions. Nevertheless, the present findings should be interpreted as preliminary because the tool was constructed from pooled study-level estimates and assessed only in a single-center retrospective cohort with a relatively high IPA/SAPA prevalence. Further multicenter external validation, local recalibration, and prospective evaluation are required before the tool can be considered for routine clinical implementation.

## Data Availability

The data analyzed in this study is subject to the following licenses/restrictions: For the protection of patient privacy, clinical data will not be disclosed. If necessary, please contact the corresponding author for access. Requests to access these datasets should be directed to Zhihai Chen: chenzhihai0001@126.com.
