# Supplementary material for: A meta-analysis-informed diagnostic stratification tool for invasive pulmonary aspergillosis in severe fever with thrombocytopenia syndrome: systematic review, meta-analysis, and single-center cohort-based assessment
Source: Front Cell Infect Microbiol. 2026 Jul 2;16:1747950. doi: 10.3389/fcimb.2026.1747950 (PMC13372647; doi:10.3389/fcimb.2026.1747950)
Supplement: Supplementary file 1 [file Table1.docx]

**Supplementary Material**

**Supplementary Methods**

**Search strategy (up to May 5, 2025)**

The search strategy was designed to maximize sensitivity for studies evaluating IPA/SAPA occurrence and related risk associations in patients with SFTS.

**① PubMed:**

("Severe Fever with Thrombocytopenia Syndrome"[Mesh]

OR "severe fever with thrombocytopenia syndrome"[Title/Abstract]

OR SFTS[Title/Abstract]

OR SFTSV[Title/Abstract]

OR "SFTS virus infection"[Title/Abstract]

OR "Bandavirus dabieense"[Title/Abstract])

AND

("Aspergillosis, Invasive"[Mesh]

OR "invasive pulmonary aspergillosis"[Title/Abstract]

OR IPA[Title/Abstract]

OR SAPA[Title/Abstract]

OR "SFTS-associated pulmonary aspergillosis"[Title/Abstract]

OR "pulmonary aspergillosis"[Title/Abstract])

AND

("Risk Factors"[Mesh]

OR "risk factor*"[Title/Abstract]

OR predictor*[Title/Abstract]

OR "associated factor*"[Title/Abstract]

OR "odds ratio"[Title/Abstract]

OR "relative risk"[Title/Abstract])

**② Web of Science:**

TS=(("severe fever with thrombocytopenia syndrome" OR SFTS OR SFTSV OR "SFTS virus infection" OR "Bandavirus dabieense")

AND

("invasive pulmonary aspergillosis" OR IPA OR SAPA OR "SFTS-associated pulmonary aspergillosis" OR "pulmonary aspergillosis")

AND

("risk factor*" OR predictor* OR "associated factor*" OR "odds ratio" OR "relative risk"))

**③ Cochrane Library:**

(MeSH descriptor: [Severe Fever with Thrombocytopenia Syndrome] explode all trees

OR ("severe fever with thrombocytopenia syndrome" OR SFTS OR SFTSV OR "SFTS virus infection" OR "Bandavirus dabieense"):ti,ab,kw)

AND

(MeSH descriptor: [Aspergillosis, Invasive] explode all trees

OR ("invasive pulmonary aspergillosis" OR IPA OR SAPA OR "SFTS-associated pulmonary aspergillosis" OR "pulmonary aspergillosis"):ti,ab,kw)

AND

(MeSH descriptor: [Risk Factors] explode all trees

OR ("risk factor*" OR predictor* OR "associated factor*" OR "odds ratio" OR "relative risk"):ti,ab,kw)

**④ Embase:**

('severe fever with thrombocytopenia syndrome'/exp

OR 'severe fever with thrombocytopenia syndrome':ti,ab,kw

OR sfts:ti,ab,kw

OR sftsv:ti,ab,kw

OR 'sfts virus infection':ti,ab,kw

OR 'bandavirus dabieense':ti,ab,kw)

AND

('invasive aspergillosis'/exp

OR 'pulmonary aspergillosis'/exp

OR 'invasive pulmonary aspergillosis':ti,ab,kw

OR ipa:ti,ab,kw

OR sapa:ti,ab,kw

OR 'sfts-associated pulmonary aspergillosis':ti,ab,kw)

AND

('risk factor'/exp

OR 'predictor variable'/exp

OR 'odds ratio'/exp

OR 'relative risk'/exp

OR 'risk factor*':ti,ab,kw

OR predictor*:ti,ab,kw

OR 'associated factor*':ti,ab,kw

OR 'odds ratio':ti,ab,kw

OR 'relative risk':ti,ab,kw)

**Additional search procedures**

Reference lists of all included articles and relevant reviews were manually screened to identify any additional eligible studies. No additional eligible studies were identified through reference-list screening. Duplicate records were removed before title/abstract screening. No formal grey-literature search was performed because the review was restricted to peer-reviewed original research articles with extractable effect estimates; conference abstracts, letters, comments, and case reports were excluded according to the predefined eligibility criteria.

**Screening criteria**

**① Inclusion criteria:**

Studies were considered eligible if they met all of the following criteria:

a. They were peer-reviewed original research articles.

b. They included patients with laboratory-confirmed severe fever with thrombocytopenia syndrome (SFTS).

c. They directly evaluated invasive pulmonary aspergillosis (IPA), SFTS-associated pulmonary aspergillosis (SAPA), or explicitly comparable Aspergillus-related pulmonary outcomes in patients with SFTS.

d. They investigated risk factors, predictors, or associated clinical characteristics for IPA/SAPA occurrence and provided extractable effect estimates, such as odds ratios (ORs), risk ratios (RRs), or sufficient raw data to calculate comparable effect measures.

e. They reported baseline or admission-related clinical, laboratory, or treatment variables in a manner that allowed comparison between patients with and without IPA/SAPA.

Patients were considered to have confirmed SFTS if the diagnosis was established by at least one of the following approaches:

a. Isolation of SFTS virus from clinical specimens;

b. Detection of SFTS virus RNA in serum or other appropriate samples;

c. A fourfold or greater increase in specific antibody titers between acute-phase and convalescent-phase paired sera.

**② Exclusion criteria**

Studies were excluded if they met any of the following criteria:

a. They were non-original publications, including reviews, editorials, letters, comments, conference abstracts, meeting reports, or case reports.

b. They were duplicate publications or reported overlapping populations without providing sufficiently independent data.

c. They were in vitro studies, animal studies, or mechanistic studies without clinical outcome data in patients with SFTS.

d. They did not directly compare patients with IPA/SAPA against those without IPA/SAPA, or did not provide extractable data relevant to IPA/SAPA risk assessment.

e. They focused primarily on broader pulmonary infection, invasive pulmonary fungal infection, genotype analysis, treatment efficacy, or non-baseline parameters without directly evaluating IPA/SAPA as the primary outcome of interest.

f. They included fewer than 20 patients or did not provide sufficient information for data extraction.

**③ Notes on study classification**

Studies using broader SAPA-oriented frameworks were retained in the primary synthesis when the article directly evaluated IPA/SAPA-related risk associations in patients with SFTS. However, these studies were interpreted cautiously because their outcome definitions were broader than those used in classic IPA-focused studies. Differences in outcome classification and predictor definitions across studies are summarized in Supplementary Tables S6 and S7.

**Supplementary Tables**

**Supplementary Table S1. Baseline characteristics of the 6 cohorts included in the systematic review and meta-analysis.**

| **Study** | **Country/region** | **Study design and period** | **Sample size (n)** | **IPA/SAPA cases, n (%)** | **Age (years)a** | **Male, n (%)** | **Outcome term** | **Key reported predictors** |
| --- | --- | --- | --- | --- | --- | --- | --- | --- |
| Dai et al. 2023 | Nanjing, China | Retrospective; May 2011–Dec 2021 | 189 | 39 (20.6%) | 63.16 ± 11.3 | 100 (52.9%) | IPA | Smoking, ICU admission, corticosteroid use, creatinine, cough, broad-spectrum antibiotic therapy |
| Hu et al. 2021 | Anhui, China | Retrospective; Mar 2015–Dec 2019 | 76 | 30 (39.5%) | 66.75 ± 9.55 | 46 (60.5%) | IPA | CD4+ T-cell count, IL-6, BNP |
| Song et al. 2023 | Shandong, China | Retrospective; Apr 2021–Aug 2022 | 67 | 22 (32.8%) | 64.7 ± 8.4 | 34 (50.7%) | IPA | Uncontrolled diabetes, CNS symptoms, platelet <40×10^9/L, CD4+ T-cell count, CD8+ T-cell count |
| Wang et al. 2024 | Wuhan, China | Retrospective; Jan 2017–Dec 2022 | 269 | 118 (43.9%) | 63.08 ± 9.70 | 167 (62.1%) | SAPA | Age, neurological complications/tremor, glucocorticoid use, ICU admission, viral load, WBC, PLT, ALB, GLB, cTnI |
| Xu et al. 2021 | Nanjing, China | Retrospective; Jan 2016–Dec 2019 | 91 | 29 (31.9%) | 62 (51–72) | 38 (41.8%) | IPA | Diabetes, cough, wheezing, vasopressor use, encephalopathy, ICU transfer |
| Yao et al. 2024 | Hubei and Anhui, China | Multicenter retrospective cohort; May 2013–Sep 2022 | 1650 | 169 (10.2%) | 61 (52–68) | 739 (44.8%) | SAPA | Advanced age, petechia, hemoptysis, tremor, low albumin, prolonged APTT, ICU admission, glucocorticoid use, IVIG, prolonged hospitalization |

Abbreviations: IPA, invasive pulmonary aspergillosis; SAPA, SFTS-associated pulmonary aspergillosis; ICU, intensive care unit; BNP, B-type natriuretic peptide; IL-6, interleukin-6; WBC, white blood cell count; PLT, platelet count; ALB, albumin; GLB, globulin; APTT, activated partial thromboplastin time; IVIG, intravenous immunoglobulin; cTnI, cardiac troponin I; CNS, central nervous system.

a Age is reported as mean ± SD or median (range / interquartile range), according to the source article.

**Supplementary Table S2. Newcastle-Ottawa Quality Assessment Scale of the 6 cohort studies.**

| **Study** | **Representativeness of sample** | **Ascertainment of exposure/data collection** | **Sample size** | **Comparability / confounding control** | **Outcome assessment** | **Statistical analysis** | **Adequate follow-up / reporting** | **Total score** |
| --- | --- | --- | --- | --- | --- | --- | --- | --- |
| Dai et al. 2023 | 1 | 2 | 1 | 1 | 1 | 1 | 1 | 8 |
| Hu et al. 2021 | 1 | 2 | 1 | 1 | 1 | 1 | 1 | 8 |
| Song et al. 2023 | 1 | 2 | 1 | 1 | 1 | 1 | 1 | 8 |
| Wang et al. 2024 | 1 | 2 | 1 | 1 | 1 | 1 | 1 | 8 |
| Xu et al. 2021 | 1 | 2 | 1 | 1 | 1 | 1 | 1 | 8 |
| Yao et al. 2024 | 1 | 1 | 1 | 1 | 1 | 1 | 1 | 7 |

Scoring shown as previously harmonized for the cohort studies included in the updated meta-analysis.

**Supplementary Table S3. Additional methodological and clinical characteristics of the included studies**

| **Study** | **Population type** | **Severe-only cohort** | **Multicenter** | **Primary outcome label** | **Broader SAPA-oriented framework used** | **Adjusted ORs available** | **Key methodological / clinical notes** |
| --- | --- | --- | --- | --- | --- | --- | --- |
| Dai et al. 2023 | Hospitalized laboratory-confirmed SFTS patients | No | No | IPA | No | Yes | Smoking-focused retrospective cohort; article reported bedside clinical, treatment, and laboratory risk factors |
| Hu et al. 2021 | Severe SFTS cohort | Yes | No | IPA | No | Yes | Severe-only cohort emphasizing immune predictors; bedside variables were limited |
| Song et al. 2023 | Hospitalized laboratory-confirmed SFTS patients from two tertiary hospitals | No | Yes | IPA | No | Yes | Two-center retrospective cohort; evaluated bedside and immune-cell predictors together |
| Wang et al. 2024 | Hospitalized SFTS cohort in a tertiary infectious disease center | No | No | SAPA | Yes | Yes | Critical-care-oriented cohort; article used a broader SAPA framework and reported both univariate and multivariable predictors |
| Xu et al. 2021 | Hospitalized SFTS patients in a tertiary hospital | No | No | IPA | No | Yes | Single-center retrospective cohort focused on clinical manifestations, imaging, and treatment features of IPA |
| Yao et al. 2024 | Multicenter hospitalized SFTS cohort | No | Yes | SAPA | Yes | Yes | Large multicenter cohort; broader SAPA-oriented framework and clinically pragmatic candidate predictors |

SAPA-oriented studies were retained in the primary synthesis when the article directly evaluated IPA/SAPA-related risk associations in patients with SFTS, but these studies were interpreted cautiously in light of broader outcome definitions.

**Supplementary Table S4. Baseline characteristics of patients in the single-center retrospective cohort for cohort-based assessment.**

| Parameters | Total  (n = 220) | Clinically ascertained IPA/SAPA  (n = 63) | Not classifiable as IPA  (n = 157) | *P* value |
| --- | --- | --- | --- | --- |
| **Demographics** |  |  |  |  |
| Gender, n |  |  |  |  |
| Female  Male | 114 (51.8%)  106 (48.2%) | 33 (52.4%)  30 (47.6%) | 81 (51.6%)  76 (48.4%) | >0.9 |
| Age, median (IQR) | 68 (59, 75) | 67 (61, 75) | 68 (59, 74) | 0.8 |
| Hospitalization (days) | 9 (6, 14) | 12 (6, 19) | 9 (6, 12) | 0.008 |
| **Underlying conditions, n** | 95 (43.2%) | 26 (41.3%) | 69 (43.9%) | 0.832 |
| Hypertensive disease | 55 (25%) | 19 (30.2%) | 36 (22.9%) | 0.343 |
| Diabetes | 32 (14.5%) | 9 (14.3%) | 23 (14.6%) | >0.9 |
| Liver disease | 4 (1.8%) | 0 (0.0%) | 4 (2.5%) | 0.580 |
| Respiratory diseases | 7 (3.2%) | 1 (1.6%) | 6 (3.8%) | 0.676 |
| Cerebrovascular disease | 15 (6.8%) | 0 (0.0%) | 15 (9.6%) | 0.007 |
| **Symptoms and signs, n** |  |  |  |  |
| **Nonspecific symptoms** |  |  |  |  |
| Fever | 212 (96.4%) | 63 (100.0%) | 149 (94.9%) | 0.2 |
| Fatigue | 160 (72.7%) | 43 (68.3%) | 117 (74.5%) | 0.4 |
| Headache | 16 (7.3%) | 6 (9.5%) | 10 (6.4%) | 0.6 |
| Dizziness | 35 (15.9%) | 13 (20.6%) | 22 (14.0%) | 0.3 |
| Myalgia | 40 (18.2%) | 9 (14.3%) | 31 (19.7%) | 0.4 |
| Lymphadenopathy and tenderness | 50 (22.7%) | 14 (22.2%) | 36 (22.9%) | >0.9 |
| Petechiae | 20 (9.1%) | 13 (20.6%) | 7 (4.5%) | <0.001 |
| **Gastrointestinal symptoms** |  |  |  |  |
| Nausea | 85 (38.6%) | 21 (33.3%) | 64 (40.8%) | 0.4 |
| Vomiting | 57 (25.9%) | 13 (20.6%) | 44 (28.0%) | 0.3 |
| Anorexia | 93 (42.3%) | 29 (46.0%) | 64 (40.8%) | 0.6 |
| Diarrhea | 76 (34.5%) | 23 (36.5%) | 53 (33.8%) | 0.8 |
| **Respiratory symptoms** |  |  |  |  |
| Cough | 42 (19.1%) | 19 (30.2%) | 23 (14.6%) | 0.014 |
| Dyspnea | 17 (7.7%) | 13 (20.6%) | 4 (2.5%) | <0.001 |
| **Central nervous system symptoms** |  |  |  |  |
| Tremor | 79 (35.9%) | 32 (50.8%) | 47 (29.9%) | 0.006 |
| Convulsion | 4 (1.8%) | 3 (4.8%) | 1 (0.6%) | 0.13 |
| Lethargy | 61 (27.7%) | 16 (25.4%) | 45 (28.7%) | 0.7 |
| **Laboratory results, median (IQR)** |  |  |  |  |
| WBC | 2 (2, 4) | 2 (1, 3) | 2 (2, 4) | 0.4 |
| NEU | 1 (1, 2) | 1 (1, 2) | 1 (1, 3) | 0.7 |
| LY | 0 (0, 1) | 0 (0, 1) | 0 (0, 1) | 0.056 |
| MO | 0 (0, 0) | 0 (0, 0) | 0 (0, 0) | >0.9 |
| EO | 0 (0, 0) | 0 (0, 0) | 0 (0, 0) | >0.9 |
| BA | 0 (0, 0) | 0 (0, 0) | 0 (0, 0) | 0.6 |
| RBC | 5 (4, 5) | 5 (4, 5) | 5 (4, 5) | 0.9 |
| HGB | 144 (132, 156) | 144 (136, 156) | 144 (130, 154) | 0.6 |
| PLT | 64 (48, 83) | 55 (44, 75) | 67 (50, 87) | 0.008 |
| MPV | 10 (10, 11) | 11 (10, 11) | 10 (10, 11) | 0.4 |
| PCT | 0 (0, 1) | 0 (0, 1) | 0 (0, 0) | <0.001 |
| CRP | 5 (1, 10) | 8 (3, 17) | 3 (1, 10) | 0.001 |
| LDH | 655 (384, 1,052) | 1,121 (535, 1,838) | 568 (358, 920) | <0.001 |
| CK | 626 (251, 1,408) | 765 (379, 2,045) | 533 (230, 1,231) | 0.010 |
| CKMB | 8 (5, 14) | 8 (5, 14) | 8 (4, 14) | 0.4 |
| ALT | 79 (51, 141) | 88 (56, 212) | 75 (50, 130) | 0.061 |
| AST | 167 (100, 336) | 293 (128, 515) | 141 (86, 281) | <0.001 |
| TBIL | 10 (8, 13) | 10 (8, 13) | 10 (8, 13) | 0.3 |
| DBIL | 3 (2, 5) | 3 (2, 5) | 3 (2, 5) | 0.2 |
| ALB | 31 (29, 34) | 32 (28, 34) | 31 (29, 34) | 0.6 |
| GLOB | 27 (25, 30) | 27 (24, 29) | 28 (26, 31) | 0.007 |
| GGT | 30 (20, 65) | 38 (21, 107) | 28 (20, 56) | 0.031 |
| ALP | 64 (49, 81) | 67 (49, 95) | 63 (50, 79) | 0.2 |
| UREA | 7 (5, 9) | 8 (5, 11) | 6 (5, 9) | 0.022 |
| CREA | 68 (55, 89) | 79 (63, 102) | 66 (52, 85) | 0.003 |
| Na | 135 (131, 138) | 134 (131, 138) | 135 (131, 138) | >0.9 |
| K | 4 (3, 4) | 4 (4, 4) | 4 (3, 4) | <0.001 |
| TT | 24 (21, 28) | 26 (21, 38) | 23 (21, 26) | 0.014 |
| APTT | 50 (43, 58) | 56 (50, 64) | 48 (42, 56) | <0.001 |
| PT | 13 (12, 14) | 13 (13, 14) | 13 (12, 13) | <0.001 |
| Fb | 2 (2, 3) | 2 (2, 3) | 3 (2, 3) | 0.3 |
| PTA | 108 (97, 122) | 101 (91, 109) | 111 (101, 126) | <0.001 |

1. n (%); Median (Q1, Q3)

2. Pearson's Chi-squared test; Wilcoxon rank sum test

3. Abbreviations: WBC: White Blood Cell, NEU: Neutrophil, LY: Lymphocyte, MO: Monocyte, EO: Eosinophils, BA: Basophils, RBC: Red Blood Cell, HGB: Hemoglobin, PLT: Platelet, MPV: Mean Platelet Volume**,​** LDH: Lactate dehydrogenase, CK: Creatine phosphokinase, CKMB: Creatine Kinase-Myocardial Band​, ALT: Alanine aminotransaminase, AST: Aspartate aminotransferase, TBIL: Total Bilirubin, DBIL: Direct Bilirubin, ALB: Albumin, GLOB: Globulin, GGT: γ-glutamyl transferase, ALP: Alkaline phosphatase, PCT: Procalcitonin, CRP: C-reactive protein, CREA: Creatinine, TT: Thrombin Time, APTT: Activated Partial Thromboplastin Time, PT: Prothrombin time, PTA: Prothrombin Activity.

4. In this table, the clinically ascertained IPA/SAPA group refers to the primary endpoint, including probable IPA and suspected only / empirically treated cases.

**Supplementary Table S5. Study-level effect estimates included in the primary pooled analyses.**

| **Predictor** | **Study** | **Total N** | **IPA/SAPA cases** | **Outcome** | **Original variable** | **Estimate type** | **Study-specific OR (95% CI)** | **Source** |
| --- | --- | --- | --- | --- | --- | --- | --- | --- |
| Age | Wang (2024) | 269 | 118 | SAPA | Age_63.5 | Adjusted | 2.28 (1.25-4.17) | Table 2 |
| Age | Yao (2024) | 1650 | 169 | SAPA | Age | Adjusted | 1.04 (1.02-1.06) | Supplementary Table S2 |
| Corticosteroid use | Dai (2023) | 189 | 39 | IPA | Corticosteroid use | Adjusted | 4.63 (1.50-14.31) | Table 2 |
| Corticosteroid use | Wang (2024) | 269 | 118 | SAPA | Glucocorticoids | Univariate | 2.11 (1.26-3.55) | Table 2 |
| Corticosteroid use | Yao (2024) | 1650 | 169 | SAPA | Glucocorticoids | Adjusted | 1.73 (1.16-2.59) | Supplementary Table S2 |
| Diabetes | Song (2023) | 67 | 22 | IPA | Uncontrolled diabetes | Adjusted | 4.00 (1.11-14.49) | Table 2 |
| Diabetes | Wang (2024) | 269 | 118 | SAPA | T2DM | Univariate | 1.83 (1.07-3.15) | Table 2 |
| ICU admission | Dai (2023) | 189 | 39 | IPA | ICU admission | Adjusted | 4.10 (1.44-11.68) | Table 2 |
| ICU admission | Yao (2024) | 1650 | 169 | SAPA | ICU admission | Adjusted | 2.58 (1.54-4.27) | Supplementary Table S2 |
| Neurological symptoms | Song (2023) | 67 | 22 | IPA | CNS symptoms | Adjusted | 1.37 (0.22-8.41) | Table 2 |
| Neurological symptoms | Wang (2024) | 269 | 118 | SAPA | Neurological complications | Adjusted | 2.33 (1.24-4.38) | Table 2 |
| Neurological symptoms | Yao (2024) | 1650 | 169 | SAPA | Tremor | Adjusted | 2.92 (1.77-4.72) | Supplementary Table S2 |

Abbreviations: IPA, invasive pulmonary aspergillosis; SAPA, SFTS-associated pulmonary aspergillosis; OR, odds ratio; CI, confidence interval; ICU, intensive care unit.

All study-specific estimates were harmonized so that OR > 1 indicates higher IPA/SAPA risk. Effect estimates were analyzed on the natural-log scale before meta-analysis. Wang (2024) contributed univariate estimates for diabetes and glucocorticoid use; other listed estimates were multivariable adjusted estimates unless otherwise specified.

Yao (2024) was corrected according to Supplementary Table S2: tremor was entered as OR 2.92 (95% CI 1.77-4.72), and ICU admission was entered as OR 2.58 (95% CI 1.54-4.27).

**Supplementary Table S6. Diagnostic frameworks for IPA/SAPA across the included studies.**

| **Study** | **Outcome term used**  **in the article** | **Study population** | **Diagnostic framework reported in the article** | **Key diagnostic elements** | **Classification in the present meta-analysis** |
| --- | --- | --- | --- | --- | --- |
| Dai et al. 2023 | IPA | Hospitalized laboratory-confirmed SFTS patients | Article-reported IPA diagnosis based on combined clinical, radiologic, and fungal evidence | Chest imaging together with fungal microbiologic or biomarker evidence | IPA-focused study |
| Hu et al. 2021 | IPA | Severe SFTS cohort | Article-reported IPA diagnosis in severe SFTS based on clinical, radiologic, and mycological assessment | Chest imaging plus fungal biomarker or microbiologic evidence in a severe-only cohort | IPA-focused study |
| Song et al. 2023 | IPA | Hospitalized laboratory-confirmed SFTS patients from two tertiary hospitals | Article-reported IPA diagnosis based on combined clinical, laboratory, and radiologic evaluation | Clinical assessment, radiologic evaluation, and study-specific mycological work-up | IPA-focused study |
| Wang et al. 2024 | SAPA | Hospitalized SFTS cohort in a tertiary infectious disease center | Broader article-reported SAPA framework used in a critical-care-oriented SFTS cohort | ICU-compatible aspergillosis assessment within a broader SAPA-oriented framework | Broader SAPA-definition study included in the primary synthesis and interpreted cautiously |
| Xu et al. 2021 | IPA | Hospitalized SFTS patients | Article-reported IPA diagnosis based on chest CT findings and galactomannan-oriented fungal work-up | Chest CT and galactomannan-based fungal evaluation | IPA-focused study |
| Yao et al. 2024 | SAPA | Multicenter hospitalized SFTS cohort | Broader multicenter article-reported SAPA framework in critically ill hospitalized patients with SFTS | Multicenter SAPA ascertainment incorporating Aspergillus-focused clinical and mycological evaluation | Broader SAPA-definition study included in the primary synthesis and interpreted cautiously |

Abbreviations: IPA, invasive pulmonary aspergillosis; SAPA, SFTS-associated pulmonary aspergillosis; GM, galactomannan; EORTC/MSGERC, European Organization for Research and Treatment of Cancer/Mycoses Study Group Education and Research Consortium.

**Supplementary Table S7. Operational definitions of the five bedside candidate predictors included in the quantitative synthesis across the included studies.**

| **Study** | **Age definition** | **Corticosteroid use definition** | **Diabetes definition** | **ICU admission definition** | **Neurological symptoms definition** | **Notes on harmonization** |
| --- | --- | --- | --- | --- | --- | --- |
| Dai et al. 2023 | Age in years | Systemic corticosteroid exposure during hospitalization | Diabetes not separately reported as a standalone exposure variable | ICU admission during hospitalization | Neurological symptoms not separately reported as a standalone exposure variable | Variables were harmonized to the review-level bedside candidate predictor set when explicitly reported |
| Hu et al. 2021 | Age reported descriptively in years | Corticosteroid use not separately reported as a standalone exposure variable | Diabetes not separately reported as a standalone exposure variable | Severe-only cohort; ICU status not analyzed as a separate bedside exposure | Central nervous system symptoms | Study-specific terminology was mapped descriptively to the review-level bedside candidate predictor set |
| Song et al. 2023 | Age in years | Corticosteroid use not separately reported as a standalone exposure variable | Uncontrolled diabetes | ICU admission not separately reported as a standalone exposure variable | Central nervous system symptoms | Uncontrolled diabetes and central nervous system symptoms were used as the closest review-level matches |
| Wang et al. 2024 | Advanced age analyzed as a continuous or article-specified age variable | Glucocorticoid use during hospitalization | Diabetes not separately reported as a standalone exposure variable | ICU admission during hospitalization | Tremor or related neurologic manifestations | Article-specific variables were mapped conservatively to the review-level bedside candidate predictor set |
| Xu et al. 2021 | Age in years | Corticosteroid use not separately reported as a standalone exposure variable | History of diabetes mellitus | ICU transfer during hospitalization | Encephalopathy | ICU transfer and encephalopathy were used as the closest review-level operational matches |
| Yao et al. 2024 | Age analyzed as a continuous or article-specified age variable | Glucocorticoid use during hospitalization | Diabetes not separately reported as a standalone exposure variable | ICU admission during hospitalization | Tremor or related neurologic manifestations | Broader multicenter SAPA-oriented reporting required conservative mapping to the review-level predictors |

Abbreviations: ICU, intensive care unit.

**Supplementary Table S8. Common-effect sensitivity analyses of the five bedside candidate predictors included in the quantitative synthesis.**

| **Predictor** | **No. of studies** | **Common-effect OR (95% CI)** | **P value** | **Direction consistent with random-effects analysis** |
| --- | --- | --- | --- | --- |
| Age | 2 | 1.04 (1.02–1.06) | <0.001 | Yes |
| Corticosteroid use | 3 | 1.99 (1.47–2.70) | <0.001 | Yes |
| Diabetes | 2 | 2.06 (1.25–3.38) | 0.004 | Yes |
| ICU admission | 2 | 2.82 (1.78–4.46) | <0.001 | Yes |
| Neurological symptoms | 3 | 2.60 (1.78–3.80) | <0.001 | Yes |

Abbreviations: OR, odds ratio; CI, confidence interval.

Common-effect models were fitted as sensitivity analyses only. Primary inference in the revised manuscript is based on random-effects models using REML with Hartung–Knapp adjustment. Direction consistency indicates whether the common-effect and primary random-effects point estimates were on the same side of the null value.

**Supplementary Table S9. Diagnostic classification and key mycological evidence used for IPA ascertainment in the single-center retrospective cohort for cohort-based assessment.**

| **Section** | **Item** | **Proven IPA (n=0)** | **Probable IPA (n=51)** | **Suspected only / empirically treated (n=12)** | **Not classifiable as IPA (n=157)** |
| --- | --- | --- | --- | --- | --- |
| Final research classification | n (% of total cohort) | 0 (0.0%) | 51 (23.2%) | 12 (5.5%) | 157 (71.4%) |
| Diagnostic / mycological evidence | Compatible chest CT | — | 51 (100.0%) | 11 (91.7%) | 0 (0.0%) |
| Diagnostic / mycological evidence | Positive serum GM | — | 40 (78.4%) | 1 (8.3%) | 0 (0.0%) |
| Diagnostic / mycological evidence | Positive BDG | — | 38 (74.5%) | 4 (33.3%) | 0 (0.0%) |
| Diagnostic / mycological evidence | Bronchoscopy performed | — | 47 (92.2%) | 5 (41.7%) | 0 (0.0%) |
| Diagnostic / mycological evidence | Positive respiratory specimen culture | — | 41 (80.4%) | 0 (0.0%) | 0 (0.0%) |
| Diagnostic / mycological evidence | Positive blood culture | — | 0 (0.0%) | 0 (0.0%) | 0 (0.0%) |
| Diagnostic / mycological evidence | Supportive NGS result | — | 2 (3.9%) | 0 (0.0%) | 0 (0.0%) |
| Diagnostic / mycological evidence | Histopathologic / sterile-site evidence | — | 0 (0.0%) | 0 (0.0%) | 0 (0.0%) |
| Diagnostic / mycological evidence | Empirically treated on clinical suspicion alone | — | 0 (0.0%) | 1 (8.3%) | 0 (0.0%) |

Abbreviations: IPA, invasive pulmonary aspergillosis; GM, galactomannan; BDG, (1-3)-β-D-glucan; NGS, next-generation sequencing.

Percentages in the first row are percentages of the total cohort (n = 220). Percentages in the evidence rows are calculated within each final research classification category.

Clinical suspicion or empiric/pre-emptive antifungal treatment alone was not considered sufficient for research classification of IPA unless accompanied by compatible radiologic findings and the required mycological or microbiological evidence.

**Supplementary Table S10. Step-by-step construction of the meta-analysis-informed three-variable diagnostic stratification score**

| **Predictor** | **No. of studies** | **Primary pooled random-effects OR (95% CI)** | **log(OR)** | **SE used for precision weighting** | **Precision weight** | **Global shrinkage factor** | **Variable-specific shrinkage factor** | **Final shrunken coefficient** | **Scaled raw points** | **Assigned points** |
| --- | --- | --- | --- | --- | --- | --- | --- | --- | --- | --- |
| Neurological symptoms | 3 | 2.60 (1.54–4.42) | 0.957 | 0.269 | 0.660 | 0.952 | 0.984 | 0.942 | 8.70 | 9 |
| ICU admission | 2 | 2.82 (0.28–28.60) | 1.037 | 1.182 | 0.034 | 0.952 | 0.954 | 0.989 | 9.14 | 9 |
| Corticosteroid use | 3 | 1.99 (0.92–4.32) | 0.689 | 0.395 | 0.306 | 0.952 | 0.967 | 0.667 | 6.16 | 6 |

Score construction summary

| **Item** | **Value** |
| --- | --- |
| Number of predictors | 3 |
| Assessment-cohort IPA/SAPA events used for global shrinkage | 63 |
| Global shrinkage formula | omega = max(0, 1 - p / n_events) |
| Global shrinkage factor | 0.952 |
| Point-scaling rule | Shrunken coefficients were linearly rescaled so that the sum of integer points equaled 24 points. |
| Total score range | 0–24 points |

Abbreviations: OR, odds ratio; CI, confidence interval; IPA, invasive pulmonary aspergillosis; SAPA, SFTS-associated pulmonary aspergillosis.

Primary pooled estimates were obtained from the primary random-effects meta-analysis using REML with Hartung–Knapp adjustment. All ORs were analyzed on the natural-log scale and harmonized so that OR > 1 indicates higher IPA/SAPA risk.

The global shrinkage factor was calculated as omega = max(0, 1 − p / n_events), where p is the number of predictors and n_events is the number of IPA/SAPA events in the assessment cohort. The variable-specific shrinkage factor was calculated as 1 − (1 − omega) × (1 − normalized precision weight).

Shrunken coefficients were linearly rescaled so that the sum of integer points equaled 24 points. Because these coefficients were derived from study-level meta-analytic estimates rather than individual participant data, the assigned points should be interpreted as a pragmatic evidence-informed weighting scheme rather than precise individual-level regression coefficients. Probability calibration was performed only during the cohort-based assessment and is reported in the main Table 2 and Figure 3; it was not used to change the assigned point values in this table.

**Supplementary Table S11. Diagnostic-classification sensitivity analysis using a stricter probable-IPA endpoint**

**A.** **Cohort-based performance under the stricter probable-IPA endpoint**

| **Analysis** | **Events** | **Non-events** | **AUC (95% CI)** | **Youden-derived probability threshold** | **Sensitivity** | **Specificity** | **PPV** | **NPV** | **Accuracy** | **Youden index** | **Brier score** |
| --- | --- | --- | --- | --- | --- | --- | --- | --- | --- | --- | --- |
| Stricter probable-IPA endpoint | 51 | 169 | 0.933 (0.899–0.967) | 0.322 | 0.882 | 0.917 | 0.763 | 0.963 | 0.909 | 0.800 | 0.106 |

**B. Observed probable IPA rate across predefined risk groups**

| **Risk group** | **n** | **Events** | **Observed rate, % (95% CI)** |
| --- | --- | --- | --- |
| Low risk | 108 | 1 | 0.9 (0.2–5.1) |
| Medium risk | 57 | 7 | 12.3 (6.1–23.2) |
| High risk | 55 | 43 | 78.2 (65.6–87.1) |

Abbreviations: IPA, invasive pulmonary aspergillosis; AUC, area under the receiver operating characteristic curve; CI, confidence interval; PPV, positive predictive value; NPV, negative predictive value.

The Youden-derived probability threshold is reported for descriptive cohort-based classification performance and should not be interpreted as a treatment-triggering threshold.

In this stricter endpoint sensitivity analysis, suspected only / empirically treated cases were reclassified as non-IPA, and only proven or probable IPA was considered an event. The updated three-variable diagnostic stratification score assigned 9 points for neurological symptoms, 9 points for ICU admission, and 6 points for corticosteroid use, with a total range of 0–24 points. Risk groups were predefined as low risk (0–9 points), medium risk (15 points), and high risk (24 points). These results represent diagnostic-classification sensitivity analysis and should not be interpreted as external validation.

**Supplementary Figure S1**


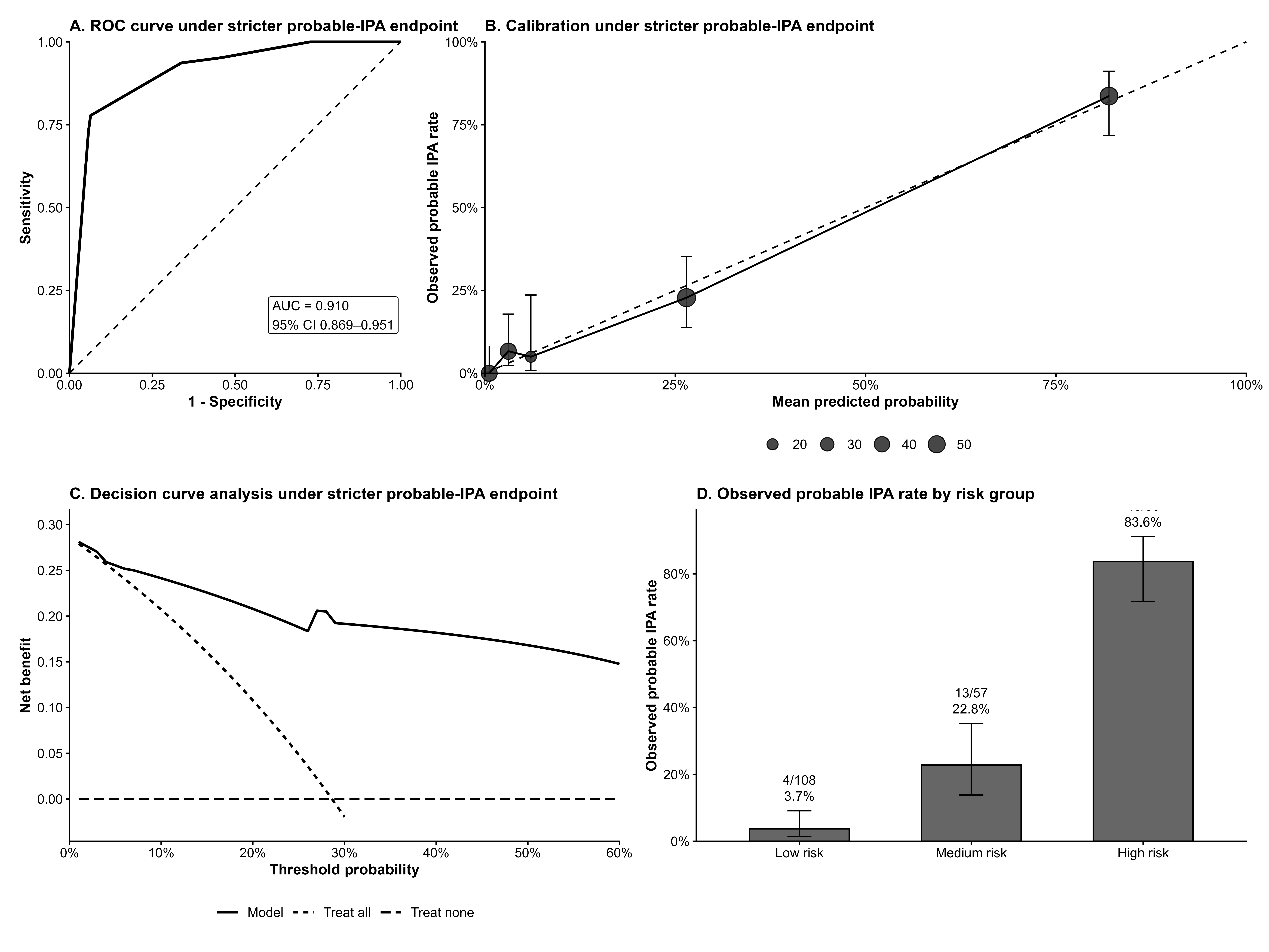


**Supplementary Figure S1. Diagnostic-classification sensitivity analysis using a stricter probable-IPA endpoint.**

(A) Receiver operating characteristic curve after reclassifying suspected only / empirically treated cases as non-IPA.

(B) Calibration plot comparing predicted probabilities with observed probable IPA rates under the stricter endpoint definition.

(C) Decision curve analysis under the stricter probable-IPA endpoint.

(D) Observed probable IPA rates across predefined low-, medium-, and high-risk groups.

In this sensitivity analysis, only proven or probable IPA was considered an event, whereas suspected only / empirically treated cases and not classifiable cases were considered non-events. Decision curve analysis should be interpreted for risk-stratified fungal diagnostic escalation only and should not be interpreted as defining a threshold for automatic antifungal treatment initiation.
